# Separate but Related: Dimensions of Healthcare Provider Social Support in Day-Treatment Oncology Units

**DOI:** 10.3389/fpsyg.2022.773447

**Published:** 2022-04-22

**Authors:** Manuela Tomai, Marco Lauriola

**Affiliations:** ^1^Department of Dynamic and Clinical Psychology, and Health, Sapienza University of Rome, Rome, Italy; ^2^Department of Social and Developmental Psychology, Sapienza University of Rome, Rome, Italy

**Keywords:** social support, healthcare, day treatment, cancer patients, scale construction and validation

## Abstract

Social support by healthcare providers has been increasingly investigated during the past decade, but studies have made different choices concerning its measurement. To evaluate how social support from a healthcare provider impacts the perceived quality of care and patient outcomes, reliable and valid instruments capable of measuring specific aspects of the construct are needed. In study 1, we tested the factor structure and the psychometric properties of a new Healthcare Provider Social Support measure (HPSS) for oncology settings. One-hundred-sixty-two patients (89 females; *M* age = 58.97, *SD* age = 13.28) from religious and government-operated hospitals completed the HPSS during day treatment. We modeled the HPSS factor structure to represent four related aspects: Emotional, Informational, Appraisal, and Instrumental social support. Study 2 preliminarily assessed the concurrent validity of the HPSS with patient perceptions of the patient-doctor relationship. Sixty-nine patients (40 females; *M* age = 53.67, *SD* age = 13.74) completed the HPPS with scales assessing perceived doctor-patient communication and patient trust in the healthcare provider. Study 1, using Exploratory Structural Equation Modeling, showed that a bifactor model had an excellent fit. The analysis supported the use of subscale scores, which were more tenable than a single total score in terms of bifactor model indices. This conclusion was also supported by greater scalability of the subscales in a Mokken Scale Analysis. Oncology patients treated in the religious hospital perceived greater Emotional, Informational, and Instrumental social support from their healthcare provider than those treated in government-operated. Study 2 showed that patient ratings of healthcare provider social support, except Instrumental, were positively correlated with better doctor communication skills and greater trust in the physician. Multiple regression analyses showed that Informational and Emotional support provided a unique contribution to building trust in the physician, controlling for the doctor’s communication skills. The study results showed that the four social support ratings were reliable and valid, sharpening the distinction between functional components in the formal healthcare system.

## Introduction

The quality of close relationships and the sense of social connectedness are reliable predictors of health and longevity (Holt-Lunstad et al., 2017; Schetter, 2017). Not surprisingly, the WHO enlisted social support networks among the most critical determinants of health (Commission on Social Determinants of Health, 2008). Social support networks perform four main functions: Emotional, Informational, Appraisal, and Instrumental. Briefly, *Emotional* support (also called affective or attachment) refers to demonstrations of love and caring, encouragement, and empathy from which one derives a sense of security (Thoits, 2011). *Informational* support is defined as providing facts or advice that may help a person solve problems (Wang et al., 2014). *Instrumental* support is intended as a form of practical, tangible help, provided through material assistance or practical tasks (Langford et al., 1997; Wang et al., 2014). Last, *Appraisal* support consists of expressions that affirm the appropriateness of acts or statements made by another (Langford et al., 1997).

Both informal and formal networks can support people affected by chronic diseases. Thus, this article refers to social support activated or required to cope with adverse life events and chronic health problems, defined as problem-oriented social support (e.g., Suurmeijer et al., 1995). Research has consistently demonstrated several benefits that medical patients receive from their informal networks (e.g., family, friends, and relatives). In conditions like diabetes, these benefits include improving emotion regulation, coping, glycemic control, and quality of life (Van Dam et al., 2005; Strom and Egede, 2012; Hill-Briggs et al., 2021). Social support also enhanced functional status and quality of life in people with heart failure, influencing treatment and health management behaviors (Graven and Grant, 2013, 2014; Bucholz et al., 2014).

In oncology patients, social support was found to address psychological problems resulting from a poor adjustment to cancer and its treatment (Rizalar et al., 2014). For example, by fostering acceptance, positive reframing of the situation, and maintaining the patient’s sense of humor, social support networks help patients be more determined in their fight against the disease and counteract helplessness-hopelessness and anxious preoccupations (Kawa, 2017; Lauriola and Tomai, 2019; Tomai et al., 2019). Moreover, several authors (Pinquart and Duberstein, 2010; Ikeda et al., 2013; Yağmur and Duman, 2016) reported significant correlations of social support with “hard” health outcomes, such as tumor initiation, cell proliferation, and life extension.

It is worth noting that social support may unintentionally hurt the patient’s wellbeing. Previous research has indicated that, contrary to caregivers’ intentions, patients who report unmet needs may perceive some forms of support as ineffective or troublesome (Breuer et al., 2017; Sebri et al., 2021). In particular, social support turns out unsupportive when the helper does not understand or effectively correspond to the recipient’s desires (Nouman and Zanbar, 2020). For example, this can happen within close relationships, when help is not sought or when support becomes controlling, oppressive, or, conversely, too superficial and neglecting the consequences of illness (Mazzoni and Cicognani, 2016; Mazzoni et al., 2017). Therefore, paying attention to the type of support a patient needs and how that support is provided will be the best way to address patients’ specific needs.

In addition to family and friends, healthcare professionals (e.g., doctors and nurses) can provide social support to patients suffering from chronic diseases, thus becoming a *formal social support network*. For instance, a supportive healthcare provider helped diabetic patients to defuse health distress and improve glycemia (Venkatesh and Weatherspoon, 2013; Wardian and Sun, 2014). Similarly, according to a recent study (Ban et al., 2021), the medical staff reduced cancer patients’ fear of illness progression by providing social support. Indeed, identifying patients at risk for psychosocial vulnerability due to low healthcare support appears to be a relevant health outcome (Usta, 2012).

How do formal social support networks work in oncology units? Are there similar functions that informal and formal networks perform for cancer patients? Healthcare social support functions have been less extensively studied than informal network ones. Nevertheless, given the increasing interest in improving healthcare quality and patient experience, constructs similar to social support functions have been used in medical settings (e.g., Donabedian, 2005; Beattie et al., 2015; Hancock et al., 2020). For instance, healthcare quality indicators include ratings of physicians’ interpersonal skills and information provision (Brédart et al., 2005a,b). Trust in oncologists reflects the patients’ beliefs about the healthcare provider’s ability to provide appropriate, reliable, and hopefully successful treatment (Müller et al., 2014). Communication is also considered a core clinical skill for establishing an excellent patient–doctor relationship (Malley and Fernández, 2010). When clinicians are better communicators, they can effectively convey information to patients, encouragement, and tangible support (Street et al., 2009). In sum, a health professional’s interpersonal competence and patient trust in the healthcare provider might be associated with one’s perception of social support. Still, they are not part of the construct as defined in psychosocial research (Barrera and Ainlay, 1983).

Where scholars have explicitly addressed healthcare social support, methodological approaches to obtaining reliable and valid measures have been varied and scattered. Previous research lacked a clear connection with the fourfold structure of the construct. For instance, some studies assessed multiple aspects of social support but used a single global score (Katz et al., 2003; Reynolds and Perrin, 2004). This choice, however, does not make fine-grained distinctions between how the healthcare provider can support the patient and improve the therapeutic relationship. Other studies focused either on emotional support (Kuuppelomäki, 2003; Wenrich et al., 2003; Ansmann et al., 2012) or informational support (Rutten et al., 2005). Underlying this approach, emotional and informational supports are viewed as functionally independent and not interchangeable. Another original approach combined two questions for each type of social support function into a single dichotomous indicator (Arora and Gustafson, 2009), assuming that healthcare provider social support could be a categorical variable. Unclear boundaries exist between informal networks’ social support functions and other proxy constructs used in medical research (e.g., communication skills).

Healthcare social support depends not only on individual actors (e.g., doctors or nurses) but also on the hospital’s mission and organizational culture. Ansmann et al. (2014), for example, emphasized the importance of hospital characteristics in fostering a better patient-doctor relationship. Hospitals in which the local culture emphasized cost control typically reported greater dissatisfaction with physicians’ interpersonal skills and information provision (Zhou et al., 2011). By contrast, non-profit hospitals imbibed in a religious culture promote a more inclusive and respectful atmosphere (Whitley, 2012). Religious hospitals have traditionally been reputed to provide a higher quality of care than government-operated hospitals (Fleming, 1981). Indeed, the organization’s charitable mission to serve and care for the person, along with a religious institutional identity, promotes the humanization of health care (e.g., Reinikka and Svensson, 2010; Calegari et al., 2015). Thus, religious hospitals can offer close and sensitive listening to the patient’s narratives and more compassionate care services, including care for the poor and vulnerable (White and Begun, 1998; White et al., 2010). Users themselves describe religious hospitals as more reliable and attractive than government-operated ones (Seemann et al., 2015). Considering this literature, a healthcare provider social support measure could also be a valuable tool to compare hospitals, outpatient clinics, particular services, or departments.

### The Present Study

The role of social support from healthcare providers is a challenging and understudied area of research. There is a need to understand how and when the support provided by health professionals may influence the quality of care and which social support function has the most significant impact on the quality-of-care cancer patients receive in oncology centers. The present study sought to develop a *Healthcare Provider Social Support* scale (HPSS), sharpening the distinction between Emotional, Informational, Instrumental, and Appraisal functions in the formal oncology care system. These domains were inspired by Cutrona and Russell (1987). However, we did not develop items related to “Opportunity for Nurturance” (i.e., the sense of being needed by others for their wellbeing) and “Social Integration” (i.e., the importance of belonging to a group that shares similar interests). These forms of social support primarily belong to informal networks (Cutrona and Russell, 1987).

To the best of our knowledge, no previous study has developed a psychometric tool that reflects the construct’s fourfold model, which proved to be valid for assessing patients’ social support from their informal network. Study 1 was designed to preliminary assess the HPSS dimensionality and reliability in a sample of patients recruited from 2 day-treatment oncology units. According to the multidimensionality of the construct, we hypothesized that at least four factors are identified in the factor analysis of a reliable item set. These factors should correspond to perceived Emotional, Informational, Instrumental, and Appraisal support. A subsequent study used an independent patient sample to assess the relationships between HPSS ratings and other related constructs in the nomological network, such as doctor communication skills and the patient’s trust in the physician. We hypothesized that greater healthcare provider support perceptions are associated with patient-centered communication and greater trust in the physician.

## Study 1: Factor Structure and Scalability of the HPSS

**Figure 1:**
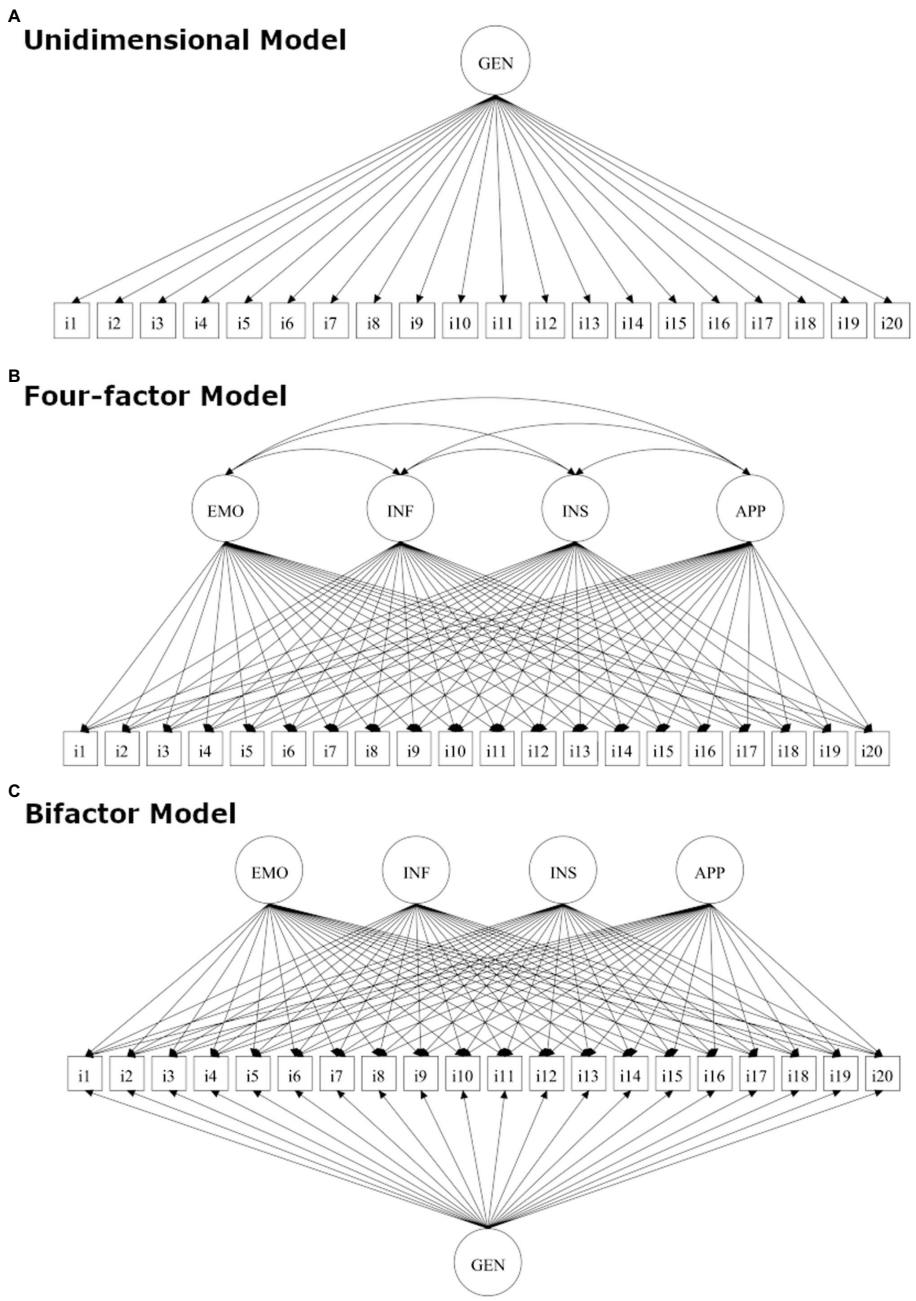
Schematic representations of the models tested in the present study: **(A)** unidimensional model; **(B)** four-factor model; and **(C)** bifactor model. EMO, emotional support; INF, informational support; INS, instrumental support; APP, appraisal support; and GEN, general support.

Investigating the factor structure of a new scale is vital to establishing its psychometric properties. Confirmatory Factor Analysis (CFA) is commonly used for this purpose. Nevertheless, CFA has recently complained about failing to account for the imperfect nature of items, as evidenced by significant item correlations with non-target constructs or cross-loadings on more than a factor (Marsh et al., 2014; Morin et al., 2016). As a result, CFA may produce inaccurate estimates of factor correlations, necessitating general or method factors to achieve an acceptable fit (Morin et al., 2016; Joshanloo et al., 2017). In the present study, we used Exploratory Structural Equation Modeling (ESEM), a new approach that overcomes the limitations of CFA (Asparouhov and Muthén, 2009). In ESEM, cross-loadings on non-target factors are allowed in addition to primary loadings on target factors. Thus, ESEM is more efficient than CFA in the estimation of complex models because it provides more accurate estimates of factor loadings and correlations, preventing artificial inflation of factor loadings on the general factor (Marsh et al., 2014; Morin et al., 2016). We further examined the HPSS dimensionality and reliability using Mokken Scaling Analysis (MSA) to provide a sensitivity analysis for ESEM results. As shown in Figure 1, we tested the following factor models, each based on different theoretical assumptions. Like previous studies (Katz et al., 2003; Reynolds and Perrin, 2004), the unifactorial model assumes that all HPSS items measure the generalized patient’s perception of the healthcare provider’s support (Figure 1A). The four-factor model is consistent with the view that patients could discriminate between ways a doctor had supported them (Figure 1B). Assuming correlated factors would imply that the four social support functions were somewhat interrelated. Small factor correlations would show that different types of support are distinct but related aspects. Moderate factor correlations would indicate substantial overlap, suggesting a hierarchical arrangement of factors. In a bifactor model, every item is targeted to load primarily on general and specific factors (Figure 1C). For example, an item describing an emotionally supportive act is hypothesized to load on a generalized support perception factor and a specific emotional support one.

As a by-product of the study, we explored whether the HPSS ratings could detect differences between two healthcare providers. In keeping with the literature (Zhou et al., 2011; Whitley, 2012; Ansmann et al., 2014), we expected that patient ratings of perceived social support on the HPSS would differ between two oncology centers reputed for religious and government-operated organizational culture, respectively. Last, we explored whether the HPSS was sensitive to how patients at different stages of disease perceived their healthcare provider as providing support for all types or just for specific kinds. This hypothesis was motivated by studies showing that a patient’s need for support from a healthcare provider may change during the cancer experience. For instance, late in the course of cancer, especially when patients perceive a poorer prognosis, there is a greater need for emotional and instrumental support from health professionals (e.g., Arora and Gustafson, 2009).

### Materials and Methods

#### Participants and Procedure

One-hundred-sixty-two consecutive patients were recruited from two oncology centers in Rome, Italy, hereafter referred to as hospitals R and G, and surveyed for this study. R was a reputed religious hospital receiving public funding; G was a government-operated hospital. All participants were patients with a confirmed cancer diagnosis receiving chemotherapy in day-treatment units. Inclusion criteria for the study were: a performance status (ECOG) of 0 or 1, age over 18 years old, written comprehension of the Italian language, and ability to fill in a paper and pencil questionnaire. Exclusion criteria were a refusal to cooperate and mental disorders due to medical conditions. The refusal rate was around 5% in both hospitals. No cases were excluded for secondary mental disorders. The ethical review board of Hospital R approved all aspects of this study. Participation was voluntary, and patients had the right to withdraw from the study. Consent was obtained before data collection. Patient characteristics are shown in Table 1.

**Table 1:** Patient characteristics (Study 1 and 2).

Characteristic	Study 1		Study 2	
*Age*	*M*	*SD*	*M*	*SD*
	58.97	13.28	59.28	12.59
*Gender*	*N*	(%)	*N*	(%)
Female	89	54.9	40	57.9
Male	73	45.1	29	42.1
Total	162	100	69	100
*Tumor site*	*N*	(%)	*N*	(%)
Stomac, colon, rectal	60	37.0	15	21.7
Female genitals	22	13.6	–	–
Breast	25	15.4	21	30.4
Skin	–	–	8	11.6
Lung	27	16.7	12	17.4
Kidney, bladder	12	7.4	1	1.4
Male genitals	8	4.9	4	5.8
Other	8	4.9	8	11.6
Total	162	100	69	100
*Stage*	*N*	(%)	*N*	(%)
I	9	5.6	31	44.9
II	16	9.9	17	24.6
III	36	22.2	6	8.7
IV	101	62.3	15	21.7
Total	162	100	162	100
*Hospital*	*N*	(%)	*N*	(%)
Government operated	60	37	69	100
Religious	102	63	–	–
Total	162	100	69	100

#### Measures

##### Healthcare Provider Social Support Scale

The HPSS administered in the present study consisted of 20 items arranged in four domains selected according to how social support functions have been operationally defined in existing scales used for informal social networks (e.g., Cutrona and Russell, 1987). The item format included the main statement (e.g., “the doctor was listening to you when you talked about your feelings”), followed by one or more illustrative examples (e.g., “pausing to talk to you or beyond the time strictly necessary to carry out his work; or leaving you time to talk to him about your fears”). The authors of this article developed the descriptive statements, which were reviewed with a medical oncologist in one of the two hospitals mentioned above. Thus, domain selection and statement generation followed what Magasi et al. (2012) considered an “etic” approach to content validity, i.e., how clinicians, researchers, or subject matter experts view the concepts to be measured. The illustrative examples were obtained from qualitative interviews with patients conducted by a hospital psychologist. Accordingly, these examples reflected an “emic” approach, i.e., they capitalized on insiders’ first-hand experience of the concept being measured (Magasi et al., 2012). The English translation of the HPSS, scoring instructions, and preliminary reference data are reported in Supplementary Materials. Patients were asked to rate their doctors’ social support behaviors during the visits or when they stay in the hospital using a five-point frequency scale (1 = Never; 2 = Rarely; 3 = Sometimes; 4 = Often; 5 = Always). Patients were instructed to resort to the examples if the main statement was unclear. Before the study, the HPSS was piloted with a small sample of patients to ensure the understandability of item content, examples, response format, and instructions. No particular problems were encountered in the pilot study.

#### Data Analysis

##### Descriptive Analysis

Before ESEM, we assessed item descriptive statistics and item-rest correlations (IRC). In classical test theory, IRC is an index of item discrimination, indicating the extent to which an item separates individuals with high and low scores on the total scale scores. IRCs >0.50, 0.30, and 0.10 are considered strong, moderate, and weak discrimination, respectively (Bechger et al., 2003). Distributional assumptions were checked using the MVN package for R (Korkmaz et al., 2019). Because Shapiro–Wilk’s test is sensitive to small departures from univariate normality, it was used in conjunction with an established rule of thumb. Accordingly, we interpreted univariate skewness and kurtosis following Kline (2015), with critical values set at 3 and 10, respectively.

##### Missing Data

One-hundred-fifty-eight patients (98%) were complete cases. Sporadic missing data were observed. The missing data pattern did not reveal configurations that would indicate missingness not at random (Little’s MCAR test = 59.41, df = 61, *p* = 0.534). Therefore, we imputed missing data based on the SPSS Expectation–Maximization procedure. There were no discernible differences between complete-case and imputed data set analyses that would invalidate the study’s conclusions. However, in ESEM analysis, we used the imputed dataset to maximize the sample size.

##### Structural Equation Modeling

ESEM analyses were conducted using *Mplus* (Version 8.4). We compared a bifactor model with the one-factor and four-factor models (Figure 1). A bifactor model is a latent structure in which each item loads on a general factor common to all items and a group factor common to some items. The general factor represents the target domain that one is most interested in (e.g., social support by a healthcare provider). The group factors represent narrow factors that explain item responses not accounted for by the general factor (e.g., information provision). According to Marsh et al. (2014), we used a Target Rotation because it provides a robust *a priori* model, allows more control over the model’s specification, and makes it easier to interpret the results. Because the HPSS uses Likert-type items, we carried out the analysis using robust least squares estimators (DWLS). This method is recommended to handle ordinal categorical data and has no distributional assumptions (Rhemtulla et al., 2012).

The model’s fit was assessed using the DWLSχ2 and other descriptive indices (Kline, 2015). CFI and TLI are incremental indices that compare the fit of the factor model to that of a null model, in which all items are assumed to be uncorrelated. CFI and TLI values >0.90 indicate acceptable fit, while values >0.95 indicate a good fit. The RMSEA measures the difference between the reproduced correlation matrix and the population correlation matrix, controlling sampling variability. An RMSEA of 0.05 or less indicates a close fit, and values up to 0.08 represent a reasonable approximation error. The 90% CI point estimate is also commonly reported to indicate the possibility of a close or exact fit.

The standardized factor loading matrix was analyzed using Dueber (2021) bifactor indices calculator package for R. It provides valuable factor and item statistics for addressing scale dimensionality and evaluating the appropriateness of the bifactor model solution. The ECV assesses the proportion of shared variance explained by each factor in the model, either general or group factors. For the general factor, ECV_G_ supports unidimensionality when values greater than 0.70 are obtained. ECV_S_ reflects the proportion of shared variance in subscale items explained by each specific factor. At the item level, IECV assesses how variance in each item can be attributed to the related variation in the general factor alone. Item unidimensionality is supported when values greater than 0.80 are obtained (Rodriguez et al., 2016). The Average Relative Parameter Bias (ARPB) cumulatively assesses the difference between an item’s loading on the general factor in the bifactor model and the corresponding loading in the unidimensional model. According to Rodriguez et al. (2016), ARPB values less than 0.15 support unidimensionality. RPB can be used to detect items for which substantial multidimensionality exists at the item level.

##### Mokken Scale Analysis

Using the Mokken package for R (van der Ark, 2007), we carried out the Mokken Scale Analysis (MSA). It is a nonparametric analog of Rasch analysis but has fewer assumptions about the shape of item characteristic curves (ICC). However, MSA assumes *local independence*, namely that participants’ responses to one item are independent of responses to other scale items if the underlying latent construct has been partialled out. This assumption was tested using W_1_ and W_3_ indices (Straat et al., 2016), which flag positive and negative locally dependent item pairs, respectively. Another assumption is *monotonicity*, namely that the probability of endorsing a specific item response category is a monotonically increasing function of the latent trait. This assumption, and the related *non-intersection* of item response curves, was tested through visual inspection of the ICC. After verifying these assumptions, we examined the *Scalability* of the HPSS total score and subscales scores. Scalability is the extent to which individual items in a scale measure the latent characteristic being measured. In MSA, Loevinger’s coefficient H test the level of Scalability for each item (H_i_) and the entire set of items that form a scale or a subscale (H_j_). A scale based on items with high H_i_ is highly scalable and likely to be unidimensional. H_j_ values greater than 0.30 are considered acceptable. According to Sijtsma and Molenaar (2002), Hj values in the range between 1.00 and 0.50 indicate strong Scalability and unidimensionality. Moderate and weak scalability ranges are between 0.49 and 0.40 and between 0.39 and 0.30, respectively.

### Results

#### Descriptive Item Analysis

As one can see from Table 2, means and SDs were comparable across items within each social support domain, showing item homogeneity. Even though the Shapiro–Wilks test was significant (all *p-s* < 0.01), the univariate skewness and kurtosis were within the acceptable limits of 3 and 10, respectively. The IRC was above the strong discrimination level for all Emotional, Informational, and Instrumental items, while it was close for Appraisal support items (Table 2). Overall, the item set proved adequate to be submitted to factor analysis.

**Table 2:** Descriptive statistics and response frequencies for the Healthcare Perceived Social Support items.

Domain	Item stem	Item descriptive statistics					Item response frequencies (%)				
	*The doctor …*	*M*	*SD*	*Sk*	*K*	*IRC*	1	2	3	4	5
Emot.	1. Comforted you by physically expressing his affection	3.90	1.31	−1.04	0.01	0.65	10.5	3.1	18.5	22.2	45.7
Emot.	2. Has been listening to you talk about your feelings	3.41	1.55	−0.43	−1.29	0.74	21.0	6.8	19.8	14.8	37.7
Emot.	3. Has shown interest and concern for your well-being	4.28	1.10	−1.51	1.51	0.49	4.3	3.1	14.8	16.0	61.7
Emot.	4. Let you know that he/she understands your mood and concerns	3.28	1.66	−0.29	−1.55	0.59	27.2	6.2	17.3	10.5	38.9
Emot.	5. Was present and heartened you in a stressful situation for you	3.37	1.59	−0.43	−1.36	0.59	24.1	4.9	17.9	16.0	37.0
Info.	6. Suggested a few actions you should take	3.20	1.67	−0.24	−1.60	0.61	29.0	6.8	14.8	13.6	35.8
Info.	7. Gave you useful information to solve your problem	3.18	1.69	−0.21	−1.63	0.64	30.9	4.9	17.3	9.9	37.0
Info.	8. Explained the pros and cons of each option you had to choose from	3.79	1.59	−0.89	−0.87	0.53	19.8	3.1	11.1	10.5	55.6
Info.	9. Made you aware of what was coming	4.48	1.06	−2.20	3.99	0.31	4.9	3.1	4.9	13.6	73.5
Info.	10. Taught you how to do something	2.91	1.73	0.06	−1.71	0.51	39.5	3.1	17.9	6.8	32.7
Instr.	11. Did some activity with you to help distract you	1.51	1.13	2.25	3.94	0.51	79.0	5.6	8.0	0.6	6.8
Instr.	12. Took you to someone who could act	1.67	1.26	1.73	1.61	0.73	74.1	4.9	9.9	3.1	8.0
Instr.	13. Helped you do something that needed to be done	2.94	1.72	0.01	−1.72	0.41	38.3	4.9	13.0	12.3	31.5
Instr.	14. Lent you or gave you something you needed	1.47	1.10	2.35	4.35	0.57	81.5	3.7	7.4	1.2	6.2
Instr.	15. Performed some tasks for you that you could not do for yourself at that time	1.59	1.20	1.93	2.47	0.59	76.5	4.9	9.3	1.9	7.4
Appr.	16. Let you know that he/she approves of the way you deal with situations	2.98	1.70	−0.02	−1.69	0.43	35.8	5.6	15.4	11.7	31.5
Appr.	17. Has expressed appreciation or respect for any of your skills or abilities	1.96	1.47	1.14	−0.32	0.39	66.0	3.1	11.7	6.8	12.3
Appr.	18. Considered you a reliable person, who can be trusted	3.94	1.43	−1.05	−0.33	0.49	12.3	5.6	14.2	12.3	55.6
Appr.	19. Treated you as an equal	4.56	0.98	−2.39	4.88	0.30	3.1	4.3	4.3	9.9	78.4
Appr.	20. Let you know that he/she appreciates you as a “person”	3.70	1.53	−0.80	−0.88	0.51	18.5	3.7	14.2	16.7	46.9

Emot., emotional support; Info., informational support; Instr., instrumental support; Appr., appraisal support; M, item mean; SD, item standard deviation; Sk, univariate skewness index; K, univariate kurtosis index; IRC, item-rest correlation; 1, never; 2, rarely; 3, sometimes; 4, often; and 5, always.

#### Exploratory Structural Equation Modeling

First, we tested the unifactor model, which assumed that all items would measure only a general perception that healthcare was supportive. This hypothesis was rejected (*χ*^2^ = 554.22; df = 170; *p* < 0.001; TLI = 0.816; CFI = 0.835; RMSEA = 0.118; SRMR = 0.127). Next, we tested the four-factor model with correlated factors, which approached the good fit for most indices (*χ*^2^ = 148.51; df = 116; *p* < 0.023; TLI = 0.977; CFI = 0.986; RMSEA = 0.042; SRMR = 0.043). The target loadings were all statistically significant for this model (all *p-s* < 0.005) and were all greater than 0.40 for Emotional (*λ*_range_ = 0.62–0.85), Informational (*λ*_range_ = 0.43–0.75) and Instrumental support (*λ*_range_ = 0.47–0.83; Table 3, Panel a). Except for item #16 (*λ* = 0.30), the Appraisal factor also loaded on items greater than 0.40. The factor loadings for appraisal support were also more heterogeneous than for Emotional, Informational, and Instrumental support (*λ*_range_ = 0.30–0.81). Although there were many significant non-target cross-loadings (i.e., 22 out of 60), these were greater than the target loading only for item #16 (Table 4, Panel a). Item simplicity (i.e., IFS > 0.70) was supported for most HPSS items. As one can see from Table 4 (Panel b), the factor correlations were significant, with a moderate effect size. The four-factor model with correlated factors expands on the one-factor model, showing that the perceived healthcare support could be broken down into separate but still related functional components. Moderate factor correlations can be explained by a general domain factor, suggesting a bifactor model approach to represent the structure of the HPSS.

**Table 3:** Standardized factor loadings and factor intercorrelations from the exploratory structural equation modeling four-factor solution of the HPSS.

Item	Panel a: factor loadings								
	Emotional		Informational		Instrumental		Appraisal		IFS
	*λ*	*p*	*λ*	*p*	*λ*	*p*	*λ*	*p*		
1	**0.80**	**(0.000)**	0.02	(0.829)	0.00	(0.975)	−0.04	(0.590)	1.00
2	**0.85**	**(0.000)**	0.05	(0.445)	0.08	(0.092)	0.03	(0.543)	0.99
3	**0.62**	**(0.000)**	0.16	(0.037)	−0.33	(0.000)	0.26	(0.000)	0.66
4	**0.66**	**(0.000)**	−0.07	(0.355)	0.10	(0.064)	0.22	(0.003)	0.87
5	**0.65**	**(0.000)**	0.23	(0.000)	0.16	(0.007)	−0.11	(0.097)	0.82
6	0.18	(0.015)	**0.58**	**(0.000)**	0.33	(0.000)	−0.09	(0.236)	0.69
7	−0.05	(0.479)	**0.71**	**(0.000)**	0.37	(0.000)	0.02	(0.840)	0.78
8	−0.06	(0.421)	**0.75**	**(0.000)**	0.11	(0.142)	−0.01	(0.843)	0.97
9	0.13	(0.116)	**0.59**	**(0.000)**	−0.31	(0.001)	0.25	(0.005)	0.66
10	0.31	(0.001)	**0.43**	**(0.000)**	0.28	(0.001)	−0.09	(0.393)	0.50
11	0.34	(0.000)	0.03	(0.738)	**0.64**	**(0.000)**	0.05	(0.630)	0.77
12	−0.13	(0.167)	0.26	(0.001)	**0.83**	**(0.000)**	0.08	(0.307)	0.88
13	−0.03	(0.728)	0.30	(0.000)	**0.47**	**(0.000)**	0.15	(0.119)	0.66
14	0.07	(0.475)	0.04	(0.748)	**0.80**	**(0.000)**	0.09	(0.439)	0.98
15	0.00	(0.983)	0.15	(0.112)	**0.74**	**(0.000)**	0.17	(0.151)	0.91
16	0.44	(0.000)	−0.04	(0.601)	0.31	(0.000)	**0.30**	**(0.004)**	0.24
17	−0.04	(0.667)	−0.12	(0.226)	0.64	(0.000)	**0.42**	**(0.001)**	0.29
18	−0.05	(0.568)	−0.05	(0.540)	−0.04	(0.615)	**0.81**	**(0.000)**	0.99
19	−0.01	(0.959)	0.35	(0.000)	−0.33	(0.000)	**0.68**	**(0.000)**	0.67
20	0.08	(0.385)	−0.19	(0.034)	0.17	(0.051)	**0.72**	**(0.000)**	0.88
*Factor*	**Panel b: factor correlations**								
	*ϕ*	*p*	*ϕ*	*p*	*ϕ*	*p*	*ϕ*	*p*	
Emotional									
Informational	0.40	(0.000)							
Instrumental	0.31	(0.000)	0.23	(0.001)					
Appraisal	0.47	(0.000)	0.36	(0.000)	0.14	(0.031)			
	Emotional		Informational		Instrumental		Appraisal		

*λ*, factor loading; *ϕ*, factor correlation; *p*, *p*-level; IFS, index of factorial simplicity, values in the 0.90s, the 80s, and the 70s are interpreted as marvelous, meritorious, and middling, respectively. Target factor loadings are shown in bold; significant non-target loadings are underlined.

**Table 4:** Standardized factor loadings and factor intercorrelations from the exploratory structural equation modeling bifactor solution of the HPSS.

	Factor loadings												
	General		Emotional		Informational		Instrumental		Appraisal		Item indexes		
	*λ*	*p*	*λ*	*p*	*λ*	*p*	*λ*	*p*	*λ*	*p*	IECV	RPB	IFSs
1	**0.56**	**(0.000)**	**0.55**	**(0.000)**	0.01	(0.872)	−0.12	(0.112)	−0.01	(0.840)	0.50	0.22	0.96
2	**0.68**	**(0.000)**	**0.62**	**(0.000)**	0.02	(0.700)	0.00	(0.959)	0.04	(0.418)	0.54	0.23	0.99
3	**0.33**	**(0.002)**	**0.65**	**(0.000)**	0.16	(0.038)	−0.08	(0.386)	0.34	(0.000)	0.16	0.87	0.74
4	**0.62**	**(0.000)**	**0.45**	**(0.000)**	−0.07	(0.299)	−0.05	(0.538)	0.16	(0.015)	0.61	0.15	0.86
5	**0.64**	**(0.000)**	**0.43**	**(0.000)**	0.17	(0.004)	0.02	(0.845)	−0.08	(0.129)	0.66	0.14	0.84
6	**0.64**	**(0.000)**	0.06	(0.338)	**0.43**	**(0.000)**	0.10	(0.288)	−0.10	(0.145)	0.67	0.09	0.89
7	**0.68**	**(0.000)**	−0.19	(0.004)	**0.58**	**(0.000)**	0.01	(0.906)	−0.03	(0.725)	0.56	0.02	0.90
8	**0.37**	**(0.000)**	0.06	(0.393)	**0.65**	**(0.000)**	0.19	(0.025)	0.05	(0.569)	0.23	0.44	0.91
9	**0.22**	**(0.092)**	0.33	(0.000)	**0.55**	**(0.000)**	−0.08	(0.521)	0.35	(0.000)	0.08	1.25	0.56
10	**0.67**	**(0.000)**	0.09	(0.222)	**0.30**	**(0.000)**	−0.08	(0.453)	−0.13	(0.121)	0.78	0.01	0.75
11	**0.80**	**(0.000)**	0.06	(0.451)	−0.07	(0.471)	**0.24**	**(0.032)**	−0.10	(0.408)	0.89	0.03	0.77
12	**0.63**	**(0.000)**	−0.13	(0.062)	0.14	(0.042)	**0.68**	**(0.000)**	−0.05	(0.480)	0.45	0.16	0.92
13	**0.54**	**(0.000)**	−0.04	(0.603)	0.21	(0.002)	**0.34**	**(0.001)**	0.08	(0.353)	0.63	0.07	0.68
14	**0.68**	**(0.000)**	−0.04	(0.541)	−0.08	(0.444)	**0.52**	**(0.000)**	−0.08	(0.472)	0.62	0.11	0.95
15	**0.67**	**(0.000)**	−0.03	(0.765)	0.03	(0.720)	**0.51**	**(0.000)**	0.01	(0.906)	0.63	0.12	0.99
16	**0.74**	**(0.000)**	0.18	(0.026)	−0.09	(0.199)	−0.07	(0.324)	**0.15**	**(0.183)**	0.89	0.06	0.33
17	**0.70**	**(0.000)**	−0.25	(0.002)	−0.21	(0.026)	0.17	(0.068)	**0.18**	**(0.221)**	0.74	0.20	0.19
18	**0.37**	**(0.002)**	−0.01	(0.871)	−0.02	(0.843)	−0.24	(0.011)	**0.71**	**(0.000)**	0.20	0.19	0.89
19	**0.13**	**(0.315)**	0.34	(0.000)	0.35	(0.000)	0.13	(0.153)	**0.79**	**(0.000)**	0.02	3.02	0.71
20	**0.49**	**(0.000)**	0.07	(0.446)	−0.16	(0.054)	−0.02	(0.858)	**0.52**	**(0.000)**	0.43	0.04	0.90

*λ*, factor loading; *p*, *p*-level. IECV, individual item explained common variance, items with large loadings on the general factor and IECV greater than 0.80 or 0.85 will typically yield a unidimensional item set that reflects the content of the general domain; RPB, individual item relative parameter bias, parameter bias less than 0.10–0.15 is acceptable and poses no serious concern regarding multidimensionality. IFSs, index of factorial simplicity for group-specific factors, values in the 0.90s, the 80s, and the 70s are interpreted as marvelous, meritorious, and middling, respectively. Target factor loadings are shown in bold; Significant non-target loadings are underlined.

The bifactor model provided a nearly perfect fit (*χ*^2^ = 121.71; df = 100; *p* = 0.069; TLI = 0.982; CFI = 0.991; RMSEA = 0.037; SRMR = 0.039) and was significantly better than the four-factor model (Δ*χ*^2^ = 28.43; df = 16; *p* < 0.05). As seen in Table 5, all items (except #9 and #19) significantly loaded on the general factor, and the coefficients were generally moderate to large (*λ*_range_ = 0.37–0.80). Except for Appraisal Support, for which only three target loadings out of five were statistically significant (*λ*_range_ = 0.52–0.79), all target loadings identified Emotional (*λ*_range_ = 0.43–0.65), Informational (*λ*_range_ = 0.30–0.65) and Instrumental support (*λ*_range_ = 0.24–0.62) factors (Table 5). As in the previous analysis, the significant cross-loadings did not threaten the simplicity of the factor solution. The index of simplicity calculated for the four group factors showed that the items maintained good purity as indicators of Emotional, Informational, Instrumental, and Appraisal support (Table 4).

**Table 5:** Descriptive statistics and intercorrelations among HPSS scores.

(a) Analysis of total score					(b) Analysis of separate subscale scores				
Item	*Hi*	*SE*	*Z-*score		Item	*Hi*	*SE*	*Z-*score	
1	0.36	(0.04)	8.26		1	0.55	(0.05)	10.86	
2	0.42	(0.04)	11.69		2	0.59	(0.04)	14.49	
3	0.30	(0.06)	5.43		3	0.46	(0.07)	6.71	
4	0.36	(0.04)	8.62		4	0.50	(0.05)	9.33	
5	0.39	(0.04)	9.63		5	0.50	(0.05)	9.28	Hj = 0.53
6	0.39	(0.04)	10.18		6	0.49	(0.05)	9.35	
7	0.37	(0.04)	9.35		7	0.51	(0.05)	10.63	
8	0.29	(0.05)	6.06		8	0.45	(0.05)	8.39	
9	0.28	(0.06)	4.44		9	0.36	(0.08)	4.31	
10	0.37	(0.04)	8.74		10	0.45	(0.06)	7.50	Hj = 0.46
11	0.55	(0.07)	8.07		11	0.46	(0.09)	5.24	
12	0.47	(0.07)	7.17		12	0.61	(0.06)	10.72	
13	0.32	(0.05)	6.49		13	0.56	(0.07)	7.53	
14	0.50	(0.09)	5.91		14	0.52	(0.10)	5.52	
15	0.48	(0.07)	6.59		15	0.52	(0.08)	6.54	Hj = 0.54
16	0.40	(0.04)	9.88		16	0.38	(0.06)	6.62	
17	0.37	(0.07)	5.44		17	0.49	(0.08)	6.16	
18	0.23	(0.05)	5.02		18	0.42	(0.05)	8.63	
19	0.29	(0.08)	3.91		19	0.35	(0.07)	4.69	
20	0.27	(0.05)	6.00	Hj = 0.36	20	0.43	(0.05)	7.89	Hj = 0.42

Hi, scalability coefficient for individual items; SE, standard error of Hi; and Hj, scalability coefficient for the total score or subscale scores.

Given that HPSS items entangled general and specific sources of variance, the following analyses were performed to determine whether total or subscale scores are supported. The general factor explained about half of the common variance in perceived social support (ECV_G_ = 51%) the remaining common variance was explained almost equally by Affective (ECV_S_ = 14%), Informational (ECV_S_ = 12%), Instrumental (ECV_S_ = 10%), and Appraisal (ECV_S_ = 13%) factors. The model ARPB was equal to 37%. Given ECV_G_ < 70% and ARPB >15%, we can conclude that multidimensionality cannot be neglected in modeling the health provider social support construct using the HPSS scale. At the item level, only four items (#10, #11, #16, and #17) with IECV >0.80 varied between participants because of variation in the general factor only. The remaining items entangled to varying degrees a portion of variance related to the general factor and a portion related to the group factor (IECV_range_ = 0.02–0.67).

Following Rodriguez et al. (2016), we compared the reliability ω coefficients separately assessed for each subscale to the corresponding hierarchical ones (ωsh) in which the general factor variance was partialled out. This comparison is to assess the unique information provided by each of the subscale scores relative to the total reliable variance in each subscale. The resulting ω coefficients were high: 0.92, 0.90, 0.89, and 0.92 for Emotional, Informational, Appraisal, and Instrumental subscales, respectively. The ωsh dropped to 0.41, 0.39, 0.59, and 0.29, respectively. For the total score, instead, the proportion of reliable variance was *ω* = 0.95, with 81% of the variance depending on the general domain factor (i.e., *ωh* = 0.81). So, using the total score would dilute too much the variance of the specific factors present in the subscales.

#### Mokken Scale Analysis

First, we evaluated the local independence assumption. For the total score (20 items), we found only one flagged item pair (i.e., #2 with #3) to be positively locally dependent; no negative local dependence was found. For the Affective and Instrumental subscales (with five items each), we found one flagged item pair to be positively locally dependent (i.e., #2 with #3 and #12 with #13, respectively). No violations were detected for the Informational and Appraisal subscales. Therefore, the local independence assumption was only sporadically violated. Second, we inspected the item characteristic curves reported in Supplementary Materials. All items comprising the total score had monotonically non-decreasing patterns, and no significant violations were detected (Supplementary Figure S1; Supplementary Table S1). The analysis of subscales revealed some violations of the monotonicity assumptions (Supplementary Figure S2); however, only three items (#2, #8, and #18 belonging to Emotional, Information, and Appraisal scores, respectively) yielded statistically significant violations (Supplementary Table S2). Taken together, the assumptions for testing item scalability are overall tenable, with a few exceptions.

Table 5 shows the scalability coefficients H_i_ for specific items under total and subscale scoring approaches. The global H_j_ for the total scale and separate subscales were also reported. Using the total score approach, five items out of 20 were poorly scalable, and the resulting scale was weakly scalable. Conversely, scoring the HPSS according to a subscale approach made most items moderately to highly scalable. The Emotional and Instrumental HPSS subscales were strong, while the Informational and Appraisal ones were moderate. The Mokken reliability coefficients were 0.88 for the total score and 0.83, 0.77, 0.83, and 0.69 for the Emotional, Informational, Instrumental, and Appraisal subscales.

#### Preliminary Criterion Validity Analyses

**Figure 2:**
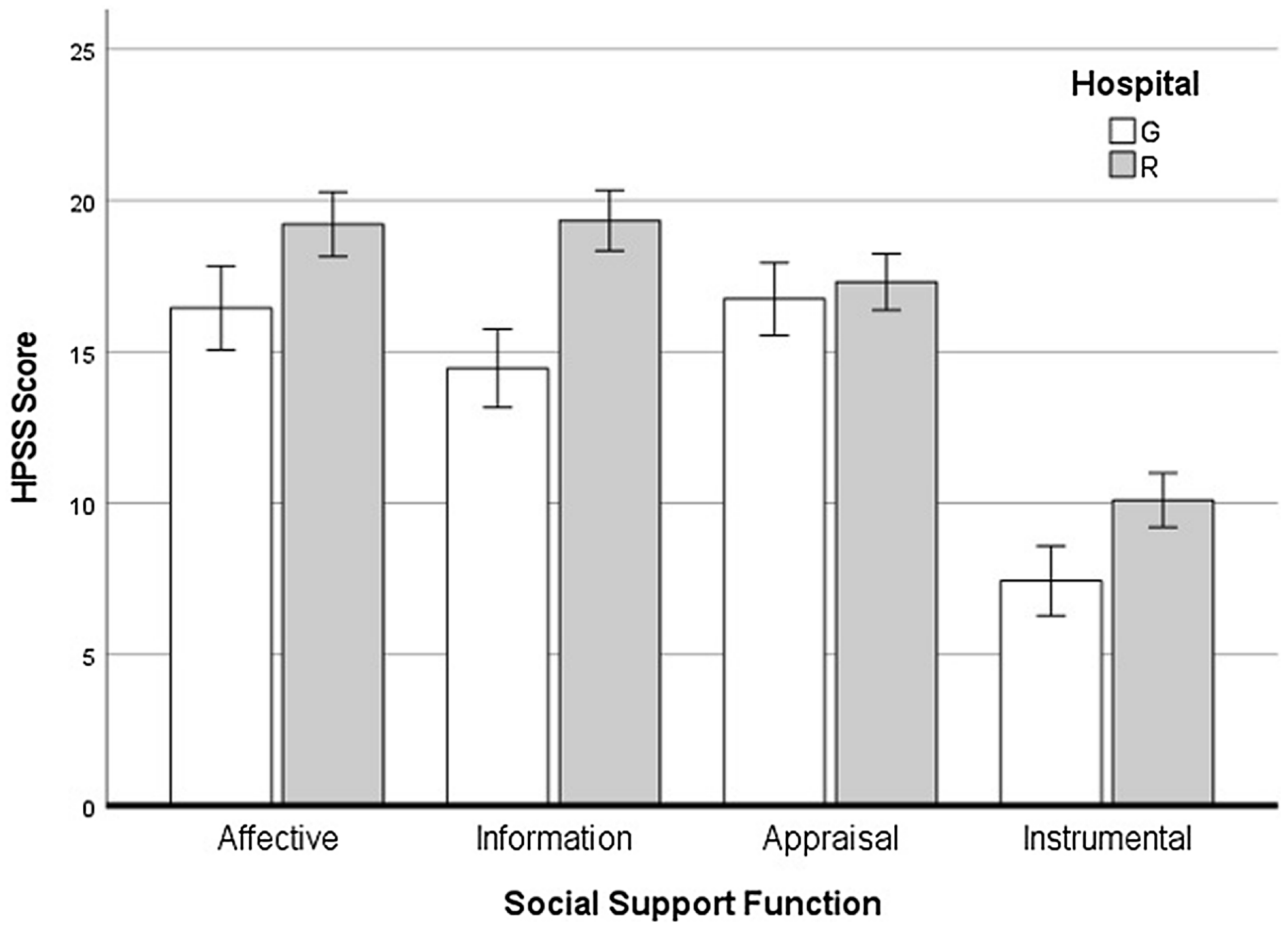
Differences between government-operated (G) and religious (R) hospitals across different social support functions.

One of the intended applications of the HPSS could be assessing the perceived level of social support provided by healthcare providers in different hospitals, outpatient clinics, services, or departments in studies of healthcare quality. We piloted this approach, involving hospitals R and G in the HPSS preliminary validation study. Patients were also stratified by disease stage (i.e., stages I–III vs. stage IV) in data analysis because the patient’s need for support from a healthcare provider may change during the cancer experience. The analysis of Emotional, Informational, Instrumental, Appraisal support by hospital and disease stage revealed statistically significant multivariate main effects for the hospital (*F* = 20.83; df = 4,157; *p* < 0.001) and hospital × support function interaction (*F* = 6.39; df = 3,155; *p* < 0.001) accounting for 12 and 11% of the variance in the combined dependent variables, respectively. Follow-up univariate analyses were examined to determine which functional components of social support accounted for the multivariate effect. Significance was determined at *p* < 0.0125 (i.e., *α* = 0.05/4) to control for familywise type 1 error. Group means are shown in Figure 2. Patients receiving treatment in the religious hospital felt more supported than those treated in the public hospital from an emotional, informative, and practical point of view. There was no difference in esteem support. Using the HPSS total score as the dependent variable, we found that patients at hospital R perceived their healthcare provider to be overall more supportive.

## Study 2: HPSS and Related Constructs

The previous study showed that the HPSS scale has a solid factorial structure and that the four social support functions can be reliably measured. However, examining only the factorial structure of the scale is not sufficient to demonstrate the validity of HPSS scores. Given that validation of a new measure is a laborious process that requires a range of empirical evidence, in this study, we surveyed an independent sample of oncology patients to take the first step in this direction. As part of criterion-related validity assessment, we aimed to establish the correlations between HPSS scores and two critical variables in the physician-patient relationship. These were the physician’s communication skills and the patient’s trust toward the healthcare provider. Recent studies have reinforced the view that patients of health professionals with better communication skills develop trusting relationships with their doctors (Chandra et al., 2018) and have better health outcomes (Howick et al., 2018; Noble, 2020). Accordingly, we expected the HPSS scores to positively correlate with the doctor’s communication skills and the patient’s trust toward the health care provider.

### Materials and Methods

#### Participants and Procedure

Sixty-nine non-consecutive patients were recruited from one oncology center in Naples, Italy. As in Study 1, all participants had a confirmed cancer diagnosis and received chemotherapy in a day-treatment unit. Inclusion and exclusion criteria were the same as in Study 1. The refusal rate was around 10%, and no cases were excluded because of secondary mental disorders. The ethical review board at Sapienza University in Rome approved the study. As a condition of participation, all patients provided their informed consent. Characteristics of patients can be found in Table 1.

#### Measures

##### Healthcare Provider Social Support Scale

Same as in Study 1.

##### Trust in the Physician Scale

This 11-item scale (TPS; Anderson and Dedrick, 1990) is one of the most widely used tools to assess patients’ trust in their physician (Müller et al., 2014). In the absence of a formal validation study for this scale in Italian, the second author of this paper translated items and instructions for use in the present study, receiving back-translation feedback from an expert bilingual professional translator. The TPS uses a five-point response scale (1 = Strongly Disagree to 5 = Strongly Agree) and yields a total score reflecting greater trust.

##### Communication Assessment Tool

This scale consists of 15 items designed to assess patient perceptions of physician interpersonal and communication skills (CAT; Makoul et al., 2007). The Italian translation of the CAT has been recently tested in an outpatient surgical clinic (Scala et al., 2016). Patients are asked to answer each item based on a single, recent physician interaction. The CAT yields a summary score reflecting the patient’s overall satisfaction.

#### Data Analysis

Pearson’s *r* was used to assess the correlations among variables. Nonlinear correlations were explored using the nlcor R package (Ranjan and Najari, 2022). The unique contribution of social support and communication scores in predicting trust was explored using linear regression analyses. The sequence of analyses and the choice of independent and dependent variables was guided by emerging findings in the correlation analyses and the reviewed literature. As in Study 1, sporadic missing data were observed (59 patients, 86%, were complete cases), and the missing data pattern was completely random (Little’s MCAR test = 472.14, df = 471, *p* = 0.477). Due to the relatively small sample size, missing data were imputed, as in Study 1.

### Results

Table 6 reports descriptive statistics, reliability coefficients, and correlations among HPSS scores, Trust in Physician, and Physician’s Communication Skills. Nonlinear correlations were virtually identical to Pearson correlations, supporting the linearity and monotonicity of all relationships. All the coefficients were positive and statistically significant. The correlations among HPSS scores were very high, reflecting a substantial proportion of shared common variance. Emotional and Informational support functions were more strongly associated with Physician’s Communication Skills than Instrumental and Appraisal ones. The total HPSS score was also strongly associated with the communication score. This result showed that physicians with better communication skills were more effective in providing social support to their patients, making them feel secure (i.e., Emotional support) and informed about their treatment and medical status (i.e., Informational support).

**Table 6:** Descriptive statistics and intercorrelations among HPSS scores, trust in physician, and physician’s communication skills.

Score (range)	*α*	*M*	*SD*	Correlations						
Emotional (1–25)	0.92	13.17	5.92	--	0.75^**^	0.75^**^	0.74^**^	0.91^**^	0.64^**^	0.76^**^
Informational (1–25)	0.88	16.06	5.47	0.75^**^	--	0.68^**^	0.74^**^	0.89^**^	0.66^**^	0.74^**^
Instrumental (1–25)	0.78	10.12	4.70	0.76^**^	0.68^**^	--	0.72^**^	0.87^**^	0.50^**^	0.57^**^
Appraisal (1–25)	0.93	14.99	6.21	0.74^**^	0.74^**^	0.72^**^	--	0.90^**^	0.61^**^	0.69^**^
HPSS Total (1–100)	0.96	54.33	19.96	0.91^**^	0.89^**^	0.87^**^	0.90^**^	--	0.68^**^	0.78^**^
TPS (15–55)	0.93	39.88	11.33	0.65^**^	0.66^**^	0.51^**^	0.62^**^	0.69^**^	--	0.60^**^
CAT (20–75)	0.96	51.10	15.05	0.76^**^	0.74^**^	0.57^**^	0.69^**^	0.78^**^	0.60^**^	--
				Emotional	Informational	Instrumental	Appraisal	Total	TPS	CAT

Pearson’s correlations reported. HPSS, health provider social support; TPS, trust in physician scale; CAT, communication assessment tool. Pearson correlations are reported below the diagonal; nonlinear correlations (italicized) are above the diagonal. *N* = 69.

** *p* < 0.01 (two-tailed).

Similarly, Emotional, Informational, and Appraisal support functions and the HPSS total score were more strongly associated with Trust in Physician than Instrumental support. Physician’s Communication Skills were also associated with the perceived trust, but the correlation was slightly lower than those assessed between trust and social support functions. These findings suggested that healthcare provider social support and doctor-patient communication are fundamental to developing a trusting relationship and therapeutic alliance with their doctors.

Although correlations in a cross-sectional study cannot prove causal relationships, the data collected are consistent with the view that Affective, Informational, Instrumental social support, and Physician’s Communication Skills could predict Trust to a different extent and better than instrumental support. We performed linear regression analyses to explore this possibility and disentangle unique social support and communication contributions. We used the HPSS scores as predictors of trust, controlling for Physician’s Communication Skills. These analyses showed that Emotional (Beta = 0.46; *t*-value = 3.02; *p* = 0.004), Informational (Beta = 0.50; *t*-value = 3.33; *p* = 0.001), and the HPSS total score (Beta = 0.59; *t*-value = 3.79; *p* < 0.001) remained statistically associated with Trust, making Physician’s Communication skills no longer significant. Both Appraisal support (Beta = 0.39; *t*-value = 2.89; *p* = 0.005) and Physician’s Communication skills (Beta = 0.32; *t*-value = 2.38; *p* = 0.021) uniquely predicted Trust scores. Confirming the correlations reported In Table 6, Instrumental support did not predict Trust score, and Physician’s Communication was the only significant predictor (Beta = 0.46; *t*-value = 3.80; *p* < 0.001). Overall, these analyses showed that Emotional and Informational healthcare supports were essential to building trusting relationships above and beyond Physician’s Communication skills.

## Discussion

In the present study, we proposed a new scale, the HPSS, to assess healthcare social support in oncology settings. The scale was designed according to a multidimensional approach, as established in psychosocial research where social networks perform Emotional, Informational, Appraisal, and Instrumental functions (Cutrona and Russell, 1987; Langford et al., 1997; Thoits, 2011; Wang et al., 2014).

Although still preliminary, the results obtained from two studies of patients recruited from different oncology day-treatment units are promising and can be summarized as follows. First, the HPSS had a multidimensional structure. Second, the HPSS subscales proved reliable and preserved a good degree of specific information. Third, we detected differences in perceived social support between patients admitted to religious and government-operated hospitals. Fourth, the Affective, Informational, and Appraisal functions were positively correlated with doctor communication skills and patient’s trust in the physician.

Regarding the factor structure, our study showed that the four-factor model had a good fit to the data, outperforming the unifactorial model. Other studies measured healthcare provider social support either as Emotional or as Informational, implicitly assuming that these functions were enough to cover the content domain and precluding comprehensive tests of construct dimensionality (Kuuppelomäki, 2003; Wenrich et al., 2003; Rutten et al., 2005; Ansmann et al., 2012). Our findings indicated that oncology patients could discriminate how a healthcare provider could support them beyond merely demonstrating love and caring, encouragement, and empathy (Thoits, 2011) or providing facts or advice (Wang et al., 2014). Instrumental and Appraisal emerged as distinct social support factors. In a doctor-patient relationship, it could be argued that providing practical, tangible help to patients (Langford et al., 1997; Wang et al., 2014) or endorsing the appropriateness of acts or statements made by patients (Langford et al., 1997) is not required. Nevertheless, even assuming this is true for all medical specialties, it is still possible that other health professionals (e.g., nurses or physical therapists) might contribute to enhancing the perceived quality of care by providing complementary functions of social support. Foreshadowing future research, one could investigate whether Instrumental and Appraisal items apply equally well to physicians and other members of a multidisciplinary oncology care team.

Our study also revealed that a general factor coexisted with the abovementioned factors. CFA has recently complained about necessitating insubstantial or inflated general factors to achieve an acceptable fit (Morin et al., 2016; Joshanloo et al., 2017). Because we used an ESEM approach, which does not produce such statistical artifacts, we are reasonably confident that the general factor represented an additional source of reliable variance. However, the general factor could be challenging to explain. Alternative interpretations are possible. On the one hand, the general factor might reflect a “true” perception that accounts for patients’ overall feelings of being supported by healthcare professionals. This interpretation is consistent with previous research considering healthcare social support as a single evaluative dimension, disregarding fine-grained distinctions between specific functions (Katz et al., 2003; Reynolds and Perrin, 2004). On the other hand, the general factor could reflect acquiescence, social desirability, or other response sets. We believe that the first interpretation is the most likely. However, we cannot rule out the second interpretation based on the present study. Thus, future research should validate the general factor against independent response-bias measures.

Although our study supported the construct validity of the HPSS, a few items showed low loadings on the target factors or lacked factorial simplicity in ESEM analysis; a few others were shown to violate Mokken scaling assumptions. As noted elsewhere (e.g., Morin et al., 2015), the inherent difficulty in producing items that perfectly reflect the constructs intended to measure could explain these defects. However, we cannot exclude other possibilities. For example, in developing the HPSS, we adopted a mostly etic approach to content validity and item generation. However, the items also reflected an emic view of patients in the illustrative examples provided below each item (see Supplementary Materials). While combining etic/emic approaches is desirable to widen the content coverage (Magasi et al., 2012), the specific item format (i.e., general statement + illustrative examples) might have increased the item complexity. Of course, defective items might be dropped out from a revision of the HPSS; however, we did not find severely biased or insubstantial items in the present study. So, we think modifying items could be more appropriate than a deletion. Notably, notwithstanding imperfections, the subscale scores preserved a non-negligible amount of reliable information and were reasonably scalable. Therefore, we recommend using subscales in clinical assessment and research applications.

The present study also made some steps toward a more robust assessment of criterion-related and concurrent validity of the HPSS scores. In keeping with previous research (White and Begun, 1998; Reinikka and Svensson, 2010; White et al., 2010; Zhou et al., 2011; Whitley, 2012; Calegari et al., 2015; Seemann et al., 2015), we expected the HPSS to be sensitive to differences in perceived support provided to patients admitted to religious or government-operated hospitals. This hypothesis was supported for Emotional, Informational, and Instrumental support, perceived higher by patients in the religious hospital. This finding implies that the HPSS could be used in quality of care research to assess and compare hospitals, outpatient clinics, particular services, or departments. As part of criterion-related validity, we also expected stage-related differences in perceived social support as suggested by previous research (Arora and Gustafson, 2009). This hypothesis was not confirmed, however. Either the patients have not changed their demands on the doctors, or the doctors may not have adapted to the patient’s changing needs. A longitudinal study would be needed to address this issue.

Construct validation is a long-term endeavor, requiring multiple studies and accumulating evidence for the instrument’s validity. Our study aimed to establish the correlations between HPSS scores and two critical variables in the physician-patient relationship as part of this process. Constructs similar to social support functions have been used in medical research (Donabedian, 2005; Brédart et al., 2005a,b; Malley and Fernández, 2010; Müller et al., 2014). From our perspective, a physician’s communication skills and trustworthiness are not social support variables; nevertheless, we expected to see positive correlations between HPSS scores and these variables. Overall, our hypotheses about the relationships of HPSS ratings with similar constructs in the nomological network were confirmed.

Beyond merely providing initial evidence of HPSS concurrent validity, the correlation pattern reported in the present study was compatible with the view that healthcare provider social support was needed to develop a trusting doctor-patient relationship, above and beyond the physician’s excellent communication abilities. Indeed, Affective and Informational scores remained statistically associated with patients’ trust in their physician, controlling for the physician’s communication skills. Not only were the patients of doctors with excellent communication skills more likely to develop trustworthy relationships (Chandra et al., 2018), but specific healthcare support functions can also have a unique role in increasing patients’ trust.

### Limitations and Future Directions

The present study is not exempt from limitations. First, the sample used was non-probabilistic and relatively small for structural equation modeling. Nevertheless, we surveyed consecutive clinical patients from different oncology units with a high response rate. So, our sample reflects the typical user of these health services. At the same time, the result of our study might not apply to other clinical populations suffering from different diseases or psychological disorders. Although no golden rule exists for establishing the minimum sample size for structural equation modeling (Kline, 2015), analyses carried out on small samples may fail to converge or provide an improper solution. In our study, we did not encounter any of these problems; therefore, we believe the sample size was sufficient, at least from a computational point of view. Of course, future studies should consider cross-validation of the factor structure on a larger sample. Second, the factors emerging from structural equation modeling need further validation. In particular, the “true” nature of the general factor needs to be clarified. To this purpose, a new data collection must include external measures of social desirability and acquiescence to rule out the possibility that the general factor captured primarily response set variance. Third, the sample size of Study 2 was somewhat limited, and the research design was cross-sectional. Therefore, caution must be exercised to avoid overgeneralizing the results and speculating on possible causal relationships between the variables involved. More research is needed with larger samples and longitudinal designs to rule out alternative interpretations of correlational evidence. Last, all variables in the study are self-reported. This characteristic of our research might have inflated the observed correlations between HPSS scores and the criteria used in the second study. Future validation studies of healthcare social support functions are needed and should compare self-reported data to data extracted from medical records or clinical test results.

### Conclusion

Our study showed that patients can discriminate well between different ways healthcare providers can support them, and the scale proposed here can measure healthcare support as a multidimensional construct. Because healthcare provider social support can soothe psychological distress resulting from a poor adjustment to chronic conditions, a multidimensional scale might help to profile which type of social support is more salient in particular healthcare services, and which is lacking. Further longitudinal studies are needed to clarify the reciprocal relationships between social support, physician–patient communication, trust, quality of care, and health outcomes.

## Data Availability Statement

The raw data supporting the conclusions of this article will be made available by the authors, without undue reservation.

## Ethics Statement

Studies involving human participants were reviewed and approved by the Hospital R Ethics Committee (Study 1) and the Ethics Review Committee for Psychological Research, Department of Social and Developmental Psychology, Sapienza University of Rome (Study 2). The Hospital R ethics committee waived the requirement for written informed consent for participation in Study 1. We obtained written informed consent from all patients participating in Study 2 as per the Ethics Review Committee for Psychological Research recommendations.

## Author Contributions

MT and ML contributed equally to the theoretical and empirical aspects of the study. All authors contributed to the article and approved the submitted version.

## Funding

This work was supported by the Sapienza Institutional Research grants (RP1181642870C00B, RM11916B7E07E816).

## Conflict of Interest

The authors declare that the research was conducted in the absence of any commercial or financial relationships that could be construed as a potential conflict of interest.

## Publisher’s Note

All claims expressed in this article are solely those of the authors and do not necessarily represent those of their affiliated organizations, or those of the publisher, the editors and the reviewers. Any product that may be evaluated in this article, or claim that may be made by its manufacturer, is not guaranteed or endorsed by the publisher.
