# Supplementary material for: Separate but Related: Dimensions of Healthcare Provider Social Support in Day-Treatment Oncology Units
Source: Front Psychol. 2022 Apr 22;13:773447. doi: 10.3389/fpsyg.2022.773447 (PMC9072867; doi:10.3389/fpsyg.2022.773447)
Supplement: Supplementary file 1 [file Table_1.pdf]

Supplementary Materials

Separate but related: Dimensions of Healthcare Provider Social Support in Day-Treatment Oncology Units

Manuela Tomai[1], Marco Lauriola[2]

[1] Department of Dynamic and Clinical Psychology, and Health, Sapienza, University of Rome

[2] Department of Social and Developmental Psychology, Sapienza, University of Rome

Table of Contents

1) **Translation of the Healthcare Provider Social Support scale (HPSS)**
2) **SPSS Syntax for Scoring of the Healthcare Provider Social Support scale (HPSS)**
3) **Reference data for standardizing the patient's scores (Data from Study 1 and 2 combined)**
4) **Supplementary Figure S1**
5) **Supplementary Table S1**
6) **Supplementary Figure S2**
7) **Supplementary Table S2**
8) **Supplementary Table S3**

1) Translation of the Healthcare Provider Social Support scale (HPSS)

Below you will find some statements that describe the relationship between patient and hospital staff. Would you please indicate how much each statement describes your perception? If the meaning of each statement seems too vague or poorly understood, you can consider the examples for each statement. (We ask you to answer by marking a cross on the box that best describes your response)

Your healthcare provider ………….

1. **Comforted you by physically expressing his affection.** (e.g., patting you on the shoulder or making an affectionate gesture).

Never	Few times	Sometimes	Often	Always

2. **Has been listening to you talk about your feelings.** (e.g., lingering to talk to you beyond the time strictly necessary to do his job; or allowing you time to talk to him about your fears).

Never	Few times	Sometimes	Often	Always

3. **Has shown interest and concern for your well-being.** (e.g., trying to figure out how you feel, as well as how you are physically or what ailments the medications are giving you).

Never	Few times	Sometimes	Often	Always

4. **Let you know that he/she understands your mood and concerns.** (e.g., telling you how he/she has felt in a difficult situation).

Never	Few times	Sometimes	Often	Always

5. **Was present and heartened you in a stressful situation for you.** (e.g., during a medical examination or while administering therapy).

Never	Few times	Sometimes	Often	Always

6. **Suggested a few actions you should take** (e.g., following a dietary regimen, informing a family member of your health condition)

Never	Few times	Sometimes	Often	Always

7. **Gave you useful information to solve your problem** (e.g., telling you what steps to take to request medical help or how to locate a specialist).

Never	Few times	Sometimes	Often	Always

8. **Explained the pros and cons of each option you had to choose from**. (e.g., by clearly explaining the advantages or disadvantages of a decision)

Never	Few times	Sometimes	Often	Always

9. **Made you aware of what was coming.** (e.g., discomfort you may have had during a medical examination or administration of therapy)

Never	Few times	Sometimes	Often	Always

10. **Taught you how to do something.** (e.g., how to dress a burn, how to expose yourself to the sun during chemotherapy).

Never	Few times	Sometimes	Often	Always

11. **Did some activity with you to help distract you.** (e.g., watch a sporting event or a live TV broadcast, read a newspaper or comment on some news together).

Never	Few times	Sometimes	Often	Always

12. **Took you to someone who could take action.** (e.g., in health care management or administration).

Never	Few times	Sometimes	Often	Always

13. **Helped you do something that needed to be done**. (e.g., meeting with a counselor, booking a medical appointment you needed to make).

Never	Few times	Sometimes	Often	Always

14. **Lent you or gave you something you needed.**

(e.g., an object, not money).

Never	Few times	Sometimes	Often	Always

15. **Performed some tasks for you that you could not do for yourself at that time.** (e.g., making a phone call, filling out a form, or any other concrete action).

Never	Few times	Sometimes	Often	Always

16. **Let you know that he/she approves of the way you deal with situations.** (e.g., agreeing with you on how you react to a stressful event).

Never	Few times	Sometimes	Often	Always

17. **Has expressed appreciation or respect for any of your skills or abilities**. (e.g., asking for an opinion or advice on something you are an expert in).

Never	Few times	Sometimes	Often	Always

18. **Considered you a reliable person, who can be trusted** (e.g., Appreciating your accuracy/accuracy in following your medication intake or diet)

Never	Few times	Sometimes	Often	Always

19. **Treated you as an equal**. (e.g., Speaking to you with "ease," avoiding using incomprehensible terms or expressions).

Never	Few times	Sometimes	Often	Always

20. **Let you know that he/she appreciates you as a "person."** (e.g., by showing you that he is comfortable talking to you, or that he is impressed by your likability).

Never	Few times	Sometimes	Often	Always

THANK YOU FOR YOUR COOPERATION

2) SPSS Syntax for Scoring of the Healthcare Provider Social Support scale (HPSS)

```
COMPUTE Emotional=sum(HPSS1 to HPSS5).
COMPUTE Information=sum(HPSS6 to HPSS10).
COMPUTE Instrumental=sum(HPSS11 to HPSS15).
COMPUTE Appraisal=sum(HPSS16 to HPSS20).
COMPUTE Total=sum(HPSS1 to HPSS20).
EXECUTE.
```

3) Reference data for standardizing the patient's scores (Data from Study 1 and 2 combined, N = 230)

Score	Min	Max	M	SD
Emotional	5	25	16.69	6.09
Informational	5	25	17.05	5.58
Instrumental	5	25	9.41	4.71
Appraisal	5	25	16.44	5.29
Total	21	100	59.54	17.43

4) **Figure S1. Non-intersection of item characteristics curves (right panels) and monotonicity plots (left panels) for the 20 items that make up the total HPSS score.**

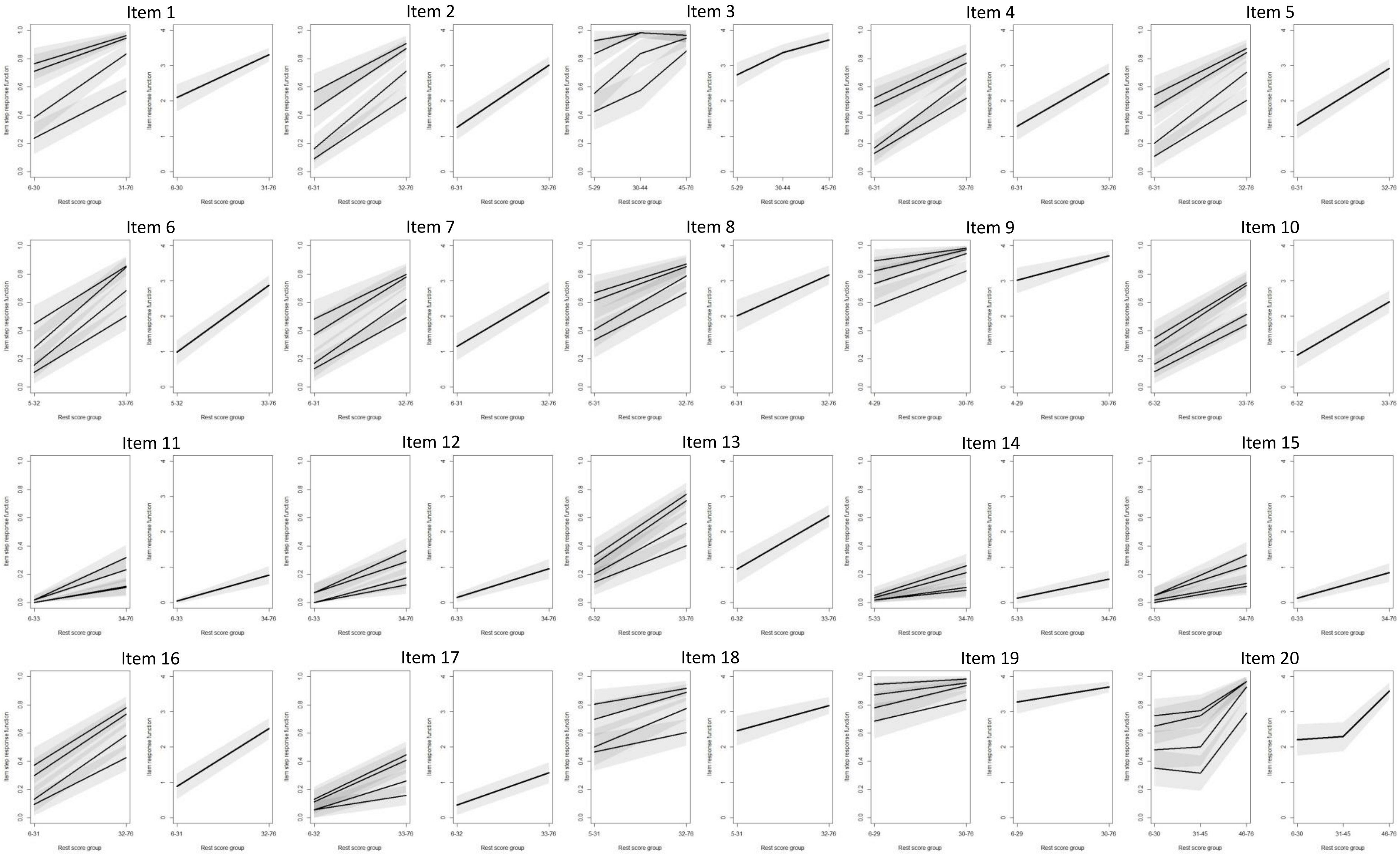

5) Table S1. Abridged output of monotonicity assessment for the 20 items that make up the total HPSS score.

	ac	vi	sig	crit
ITEM 1	4	0	0	0
ITEM 2	4	0	0	0
ITEM 3	12	0	0	0
ITEM 4	4	0	0	0
ITEM 5	4	0	0	0
ITEM 6	4	0	0	0
ITEM 7	4	0	0	0
ITEM 8	4	0	0	0
ITEM 9	4	0	0	0
ITEM 10	4	0	0	0
ITEM 11	2	0	0	0
ITEM 12	2	0	0	0
ITEM 13	4	0	0	0
ITEM 14	4	0	0	0
ITEM 15	3	0	0	0
ITEM 16	4	0	0	0
ITEM 17	4	0	0	0
ITEM 18	4	0	0	0
ITEM 19	4	0	0	0
ITEM 20	12	1	0	20

Legend: ac = active comparisons, vi = violations, sig = number of significant violations, crit = crit score

Note: A Crit score less than 40 is not evidence of model misfit. A Crit score greater than 80 is strong evidence of model misfit.

6) **Figure S2. Non-intersection of item characteristics curves (right panels) and monotonicity plots (left panels) for the items that make up the Emotional (1-5), Information (6-10), Instrumental (11-15), and Appraisal (16-20) HPSS subscales.**

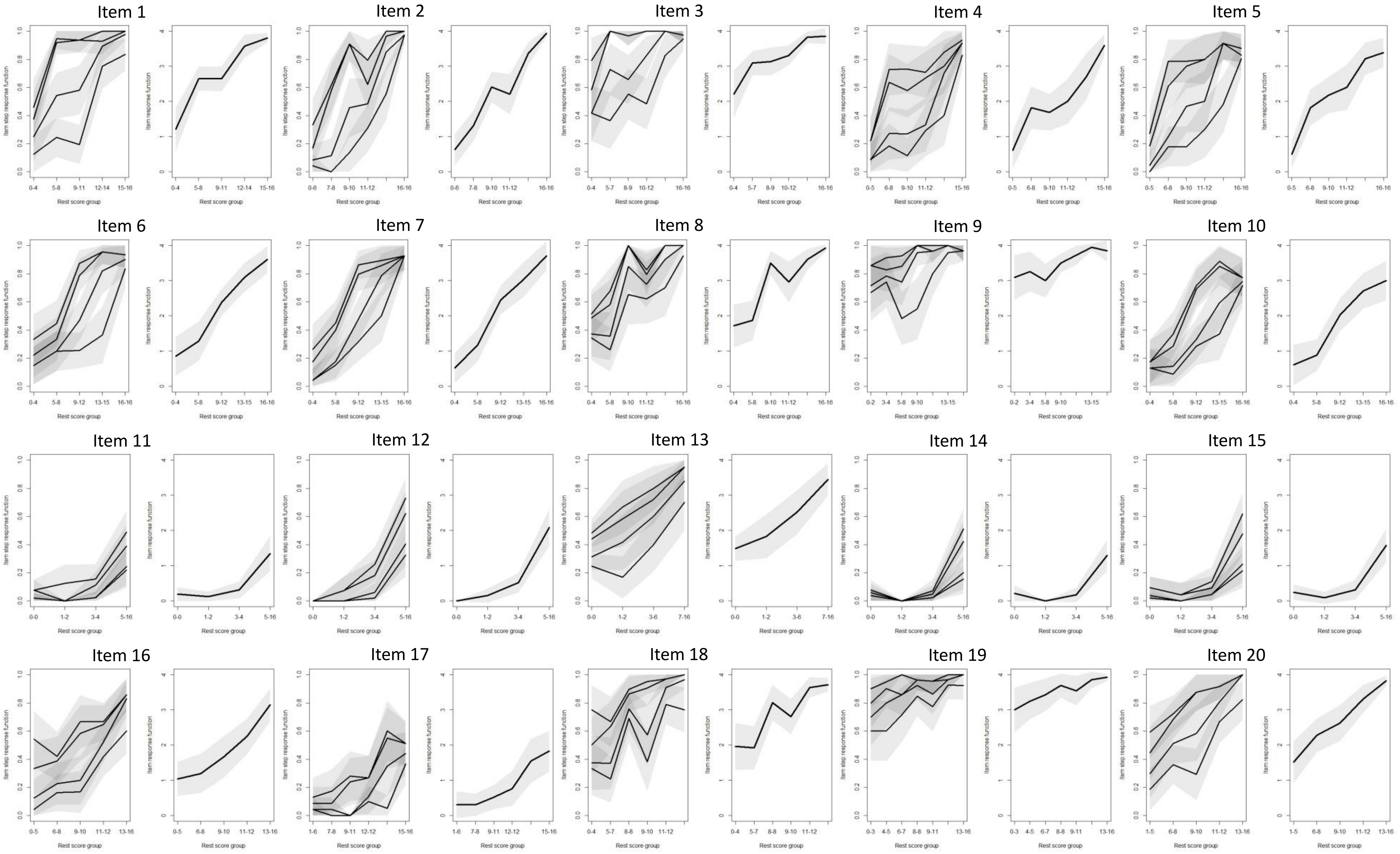

7) Table S2. Abridged output of monotonicity assessment for the items that make up the Emotional (1-5), Information (6-10), Instrumental (11-15), and Appraisal (16-20) HPSS subscales.

	ac	vi	sig	crit
ITEM 1	29	1	0	1
ITEM 2	42	3	1	62
ITEM 3	40	5	0	26
ITEM 4	60	2	0	8
ITEM 5	55	3	0	14
ITEM 6	40	0	0	0
ITEM 7	40	0	0	0
ITEM 8	33	4	1	74
ITEM 9	59	13	0	85
ITEM 10	40	3	0	28
ITEM 11	18	1	0	16
ITEM 12	8	0	0	0
ITEM 13	24	1	0	9
ITEM 14	16	4	0	44
ITEM 15	20	3	0	22
ITEM 16	40	1	0	21
ITEM 17	50	7	0	30
ITEM 18	50	5	1	80
ITEM 19	59	5	0	26
ITEM 20	28	1	0	11

Legend: ac = active comparisons, vi = violations, sig = number of significant violations, crit = crit score

Note: A Crit score less than 40 is not evidence of model misfit. A Crit score greater than 80 is strong evidence of model misfit.
